# Consequences of NMDA receptor deficiency can be rescued in the adult brain

**DOI:** 10.1038/s41380-020-00859-4

**Published:** 2020-08-17

**Authors:** Catharine A. Mielnik, Mary A. Binko, Yuxiao Chen, Adam J. Funk, Emily M. Johansson, Katheron Intson, Nirun Sivananthan, Rehnuma Islam, Marija Milenkovic, Wendy Horsfall, Ruth A. Ross, Laurent Groc, Ali Salahpour, Robert E. McCullumsmith, Shreejoy Tripathy, Evelyn K. Lambe, Amy J. Ramsey

**Affiliations:** 1 Department of Pharmacology & Toxicology, University of Toronto, Toronto, ON M5S 1A8 Canada; 2 Department of Physiology, University of Toronto, Toronto, ON M5S 1A8 Canada; 3 Krembil Centre for Neuroinformatics, Centre for Addiction and Mental Health, Department of Psychiatry, University of Toronto, Toronto, ON M5T 1L8 Canada; 4 Department of Neurosciences, University of Toledo, Toledo, OH 43614 USA; 5 Interdisciplinary Institute for NeuroScience (IINS) CNRS, Université Bordeaux Segalen, 33000 Bordeaux, France; 6 Department of OBGYN, University of Toronto, Toronto, ON M5G 1E2 Canada; 7 Department of Psychiatry, University of Toronto, Toronto, ON M5T 1L8 Canada; 8 Present Address: University of Pittsburgh School of Medicine, Pittsburgh, PA 15213 USA

**Keywords:** Neuroscience, Autism spectrum disorders

## Abstract

N-methyl-D-aspartate receptors (NMDARs) are required to shape activity-dependent connections in the developing and adult brain. Impaired NMDAR signalling through genetic or environmental insults causes a constellation of neurodevelopmental disorders that manifest as intellectual disability, epilepsy, autism, or schizophrenia. It is not clear whether the developmental impacts of NMDAR dysfunction can be overcome by interventions in adulthood. This question is paramount for neurodevelopmental disorders arising from mutations that occur in the *GRIN* genes, which encode NMDAR subunits, and the broader set of mutations that disrupt NMDAR function. We developed a mouse model where a congenital loss-of-function allele of *Grin1* can be restored to wild type by gene editing with Cre recombinase. Rescue of NMDARs in adult mice yields surprisingly robust improvements in cognitive functions, including those that are refractory to treatment with current medications. These results suggest that neurodevelopmental disorders arising from NMDAR deficiency can be effectively treated in adults.

## Introduction

Greater than 1% of children are born with a neurodevelopmental disorder [1], including diagnoses of autism spectrum disorder and pervasive developmental delay [2]. Until recently, most children with global developmental delay were not given a more specific diagnosis that could predict treatment or long-term prognosis. Whole-exome sequencing has revolutionised diagnostic assessment and has identified hundreds of genes that can cause intellectual disability and developmental delay through transmitted and de novo variants [3].

Through whole-exome sequencing, a new syndrome called *GRIN* disorder has been identified that is caused by mutations in one of the seven *GRIN* genes that encode subunits for N-methyl-D-aspartate-type glutamate receptors (NMDARs). Deleterious missense and nonsense variants in *GRIN1*, *GRIN2A-D*, and *GRIN3A-B* cause encephalopathies that are sometimes first diagnosed as intellectual disability, global developmental delay, epilepsy, autism, and/or schizophrenia [4]. The variants are often de novo heterozygous mutations that act as dominant negatives to reduce NMDAR function, although some variants lead to a gain-of function by altered channel gating properties [4]. Regardless of the nature of the mutation, patients with these deleterious variants have a similar syndrome of intellectual disability, and additional symptoms such as epilepsy, autism, cortical visual impairment, and movement disorders [4].

The identification of pathogenic variants in a *GRIN* gene allows for target-directed pharmacological treatments where approved drugs are available, but gene editing may ultimately be the most effective method to treat neurodevelopmental disorders. The timing of intervention remains a question for the future application of gene editing towards neurodevelopmental disorders. It has been assumed that intervention should occur as early in development as is medically feasible, that waiting risks irremediable damage, and that adults with these conditions are beyond the reach of medical treatment to improve cognitive function. However, these assumptions have not been stringently tested. Currently, there are many adults with these disorders that might also benefit from gene therapy, and it is unknown whether the developmental consequences of disease-causing variants can be reversed in adulthood.

The ability to reverse developmental insults is likely to depend on the nature of the insult. For example, while adult rescue of Rett syndrome gene *Mecp2* in mice reversed several phenotypes [5], adult rescue of *Shank3* in mice showed a more selective improvement to social behaviours [6]. Thus, it is conceivable that developmental insults to the NMDAR system cannot be overcome with adult intervention, considering the central role of this receptor. Indeed, NMDARs are required for the proper connectivity of developing sensory circuits in the thalamus and cortex [7–9], for the establishment of both inhibitory [10] and excitatory [11] synapses, and for the patterning of neuron dendritic arborizations [12].

Since there is strong evidence that NMDARs participate in many aspects of neurodevelopment, we asked whether developmental consequences of NMDAR deficiency could be reversed in adult mice. Specifically, we asked whether adult intervention could improve cognitive functions, since intellectual disability is a core symptom of patients with *GRIN* disorders. *GRIN1* encodes the essential subunit GluN1 that is present in all NMDARs, and null mutations of *GRIN1* are lethal in humans [13] and in mice [7, 14]. Considering the well-established role of these receptors in development and synapse refinement, it would be predicted that NMDAR deficiencies caused by *GRIN1* mutations, in particular, would be refractory to adult intervention.

To address whether developmental insults to the NMDAR system can be overcome with adult intervention, we developed a mouse model with a congenital global loss-of-function allele of *Grin1*, that could be globally genetically restored, temporally, by gene editing with Cre recombinase. We found that *Grin1* expression was restored in adulthood, and molecular analysis, cellular function and cognitive functions were quantified as outputs to measure the ability to reverse intellectual disability. Strikingly, we discovered that plasticity at the cellular, synaptic, and behavioural level was evident in the cortex. Furthermore, this rescue of cognitive ability was reproduced in a separate adult cohort and was maintained over a longer recovery time. This study suggests that plasticity of cognitive circuits extends well into adulthood, and that there is an inherent ability to upregulate NMDAR activity and to normalise cognitive outputs.

## Results

### Generation of mice with a reversible *Grin1* deficiency

**Fig. 1:**
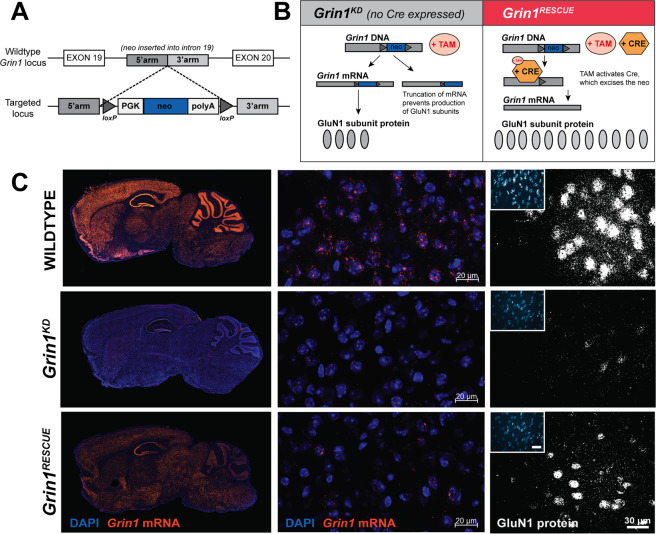
Generation and molecular characterisation of the *Grin1* inducible-rescue mouse line. **a** Targeting construct schematic and recombination events at the *Grin1* locus in the generation of the *Grin1*^*flneo/flneo*^ mouse model. PGK, PGK promotor; neo, neomycin selection cassette; polyA, polyadenylation sequence. **b** Schematic of expected molecular events at the *Grin1* locus. **c** Visualisation of *Grin1* mRNA in mouse sagittal sections (20 µm) via fluorescent in situ hybridisation using *Grin1* probes *(left: whole brain; middle: 20× micrograph of prefrontal cortex)*. At right, sagittal sections (20 µm) of prefrontal cortex visualising GluN1 protein expression via fluorescent immunohistochemistry with a rabbit anti-GluN1 antibody (in-house, 1:200; secondary anti-rabbit Alexa 568) in WT, *Grin1*^*KD*^, and *Grin1*^*RESCUE*^ mice.

To directly answer whether the developmental consequences of NMDAR deficiency could be rescued in adults, we generated mice with a reversible hypomorphic mutation in *Grin1*, the essential subunit of all NMDARs. Our previous studies, with a similar mouse line, showed that a 90% knockdown of functional NMDARs is achieved through the targeted insertion of a *neo* cassette in an intron of the *Grin1* gene [15]. In the new mouse line, we added *lox*P sites flanking the *neo* cassette to allow for inducible excision of the mutation, so that Cre recombinase could restore the locus to wild type in a conditional manner (Fig. 1a, b).

**Fig. 2:**
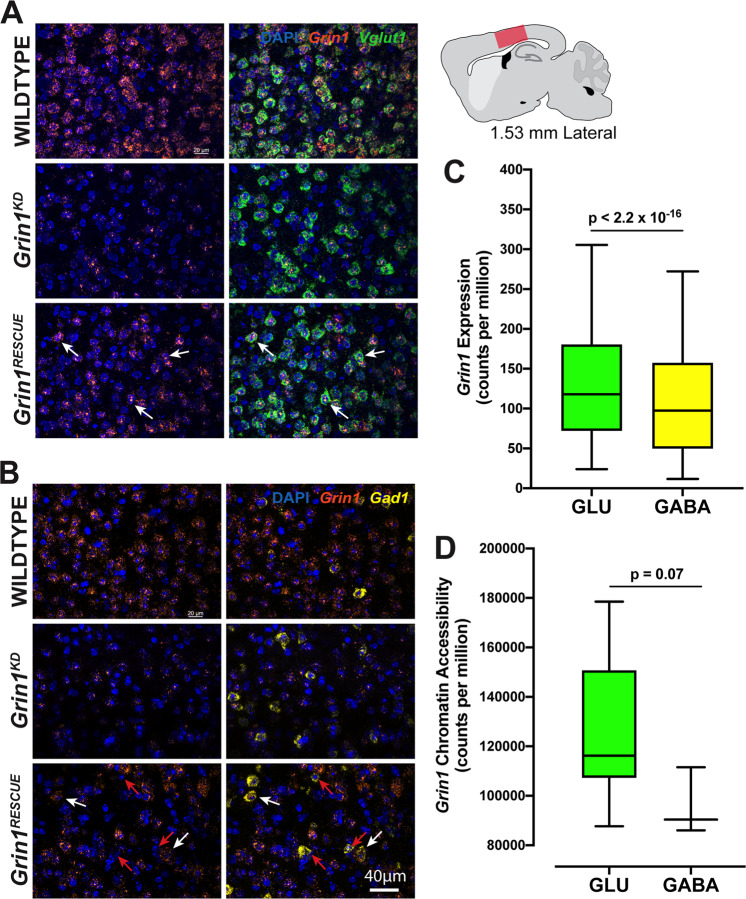
*Grin1* gene expression levels and chromatin accessibility of the adult mouse somatosensory cortex. *Grin1* mRNA expression in *Vglut*+ (**a**) and *Gad1*+ (**b**) cells in the adult mouse somatosensory cortex (1.53 mm lateral from midline). *Grin1* (orange), *Vglut1* (green) and *Gad1* (yellow) mRNA was visualised in mouse sagittal sections (20 µm) with fluorescent in situ hybridisation in WT, *Grin1*^*KD*^, and *Grin1*^*RESCUE*^ mice. Solid white arrows indicate cells with *Grin1* expression, red arrows indicate cells without *Grin1* expression. **c** *Grin1* gene expression levels and **d** *Grin1* chromatin accessibility in wild-type adult mouse visual cortex glutamatergic (*Vglut1*+) and GABAergic (*Gad1*+) cells. Data are from publicly-accessible single-cell transcriptomics data [16] and pooled cell type-specific ATAC-seq data [18] provided by the Allen Institute for Brain Science. Transcriptomic data are quantified as counts per million reads sequenced (CPM) and are based on exonic reads only. ATAC-seq data are quantified as counts per million nucleotides in locus. To aid visualisation, only cells up to the 95th percentile of *Grin1* expression or coverage are shown. Data shown as box and whisker plots, 5–95 percentile, Wilcoxon rank-sum test between GLU and GABA.

**Fig. 3:**
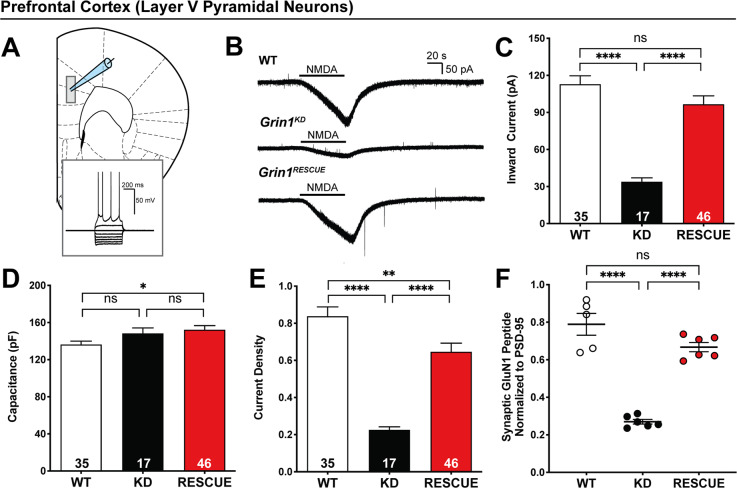
NMDAR currents and synaptic GluN1 peptide levels are restored in the mPFC of *Grin1*^*RESCUE*^ mice. **a** Schematic of mPFC with whole cell patch clamp recording from layer V adapted from [48]. Inset: electrophysiological signature of *Grin1*^*RESCUE*^ layer V pyramidal neuron. **b** Representative traces in voltage clamp (−75 mV) showing prefrontal response to NMDA across the three genotypes (number of layer V pyramidal neurons shown, 5–6 mice per genotype). **c** Quantification of peak amplitude of prefrontal NMDAR-elicited currents. One-way ANOVA, effect of genotype, *F*_2,95_ = 22, *p* < 0.0001, Bonferroni post hoc. **d** Capacitance of prefrontal layer V pyramidal neurons. One-way ANOVA, Bonferroni post hoc. **e** Current density of prefrontal NMDA-elicited currents. One-way ANOVA, effect of genotype, *F*_2,95_ = 3.6, *p* = 0.03, Bonferroni post hoc. **f** Synaptic GluN1 peptide (IVNIGAVLSTR) levels in the PFC. One-way ANOVA, effect of genotype, *F*_2,14_ = 64.76, *p* < 0.0001, Bonferroni post hoc. All data shown as mean ± SEM, *p < 0.05; **p < 0.01; ***p < 0.001; ****p < 0.0001, ns not significant.

**Fig. 4:**
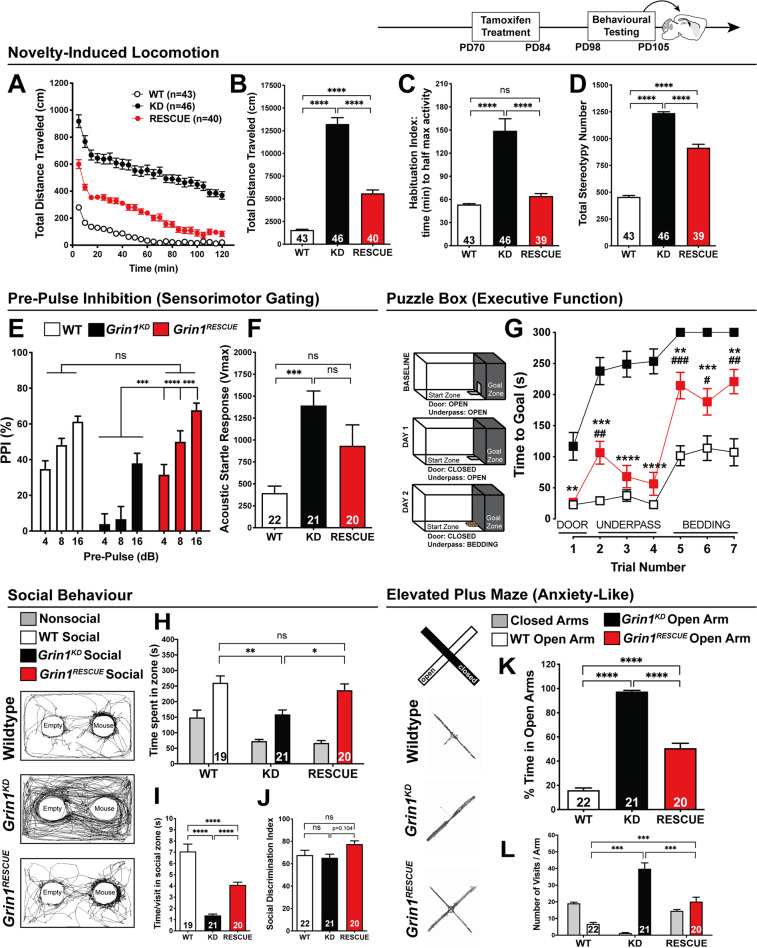
Cognitive function rescued with adult intervention, while other behaviours show intermediate improvement. All behaviours were measured in WT, *Grin1*^*KD*^, and *Grin1*^*RESCUE*^ mice starting at PD98. **a** Time course and **b** total distance travelled (cm) in open field (OF) test. One-way ANOVA (total, effect of genotype *F*_2,126_ = 156.3, *p* < 0.0001), Bonferroni post hoc. **c** Habituation index (time to reach half maximal activity) in OF test. One-way ANOVA, effect of genotype, *F*_2,125_ = 29.38, *p* < 0.0001, Bonferroni post hoc. **d** Stereotypy number in OF test. One-way ANOVA, effect of genotype, *F*_2,126_ = 382.7, *p* < 0.0001, Bonferroni post hoc. **e** Percent inhibition of startle response (pre-pulse inhibition, PPI). Shown on graph, *Grin1*^*KD*^ vs*. Grin1*^*RESCUE*^, two-way ANOVA, effect of genotype—*F*_2,60_ = 16.65, *p* < 0.0001; genotype × decibel—*F*_4,120_ = 3.526, *p* = 0.0093, Bonferroni post hoc. **f** Acoustic startle response (ASR). One-way ANOVA, effect of genotype, *F*_2,60_ = 8.913, *p* = 0.0004, Bonferroni post hoc. **g** Time to reach goal zone (seconds; max 300 s) measured in puzzle box paradigm. Shown on graph, ^#^WT vs. *Grin1*^*RESCUE*^; *Grin1^*KD*^ vs. *Grin1*^*RESCUE*.^ Two-way ANOVA, effect of genotype: *F*_2,64_ = 66.98, *p* < 0.0001, Bonferroni post hoc. *n* values: WT—21, *Grin1*^*KD*^—25, *Grin1*^*RESCUE*^—21. **h** Total time spent, **i** time per visit and **j** social discrimination index in zones during 10 min modified three chamber social test. One-way ANCOVA, there is an effect of genotype after controlling for locomotor activity: (time in social zone) *F*_2,59_ = 4.707, *p* = 0.013, (time/visit) *F*_2,59_ = 4.424, *p* = 0.016, (discrimination index) *F*_2,59_ = 3.161, *p* = 0.050, Bonferroni post hoc. **k** % time spent in open arms and **l** number of visits per arm in the elevated plus maze (EPM). One-way ANCOVA, there is an effect of genotype after controlling for locomotor activity: (% time spent in open arms) *F*_2,59_ = 280.024, *p* < 0.001, (number of visits in the open arm) *F*_2,59_ = 134.631, *p* < 0.001, Bonferroni post hoc. All data shown as mean ± SEM, *p < 0.05; **p < 0.01; ***p < 0.001; ****p < 0.0001, ns not significant.

We intercrossed these mice with Rosa26-CreERT2 mice that ubiquitously express a tamoxifen-inducible Cre recombinase. We first identified the tamoxifen regimen that comprehensively induced Cre activity throughout the brain using a Cre-reporter line (Rosa26-dTomato: Supplementary Fig. 1). We then administered tamoxifen to all genotypes of mice at either postnatal day (PD) 21, 42, or 70 and measured biochemical and behavioural endpoints at either PD98 or PD105 (2-week treatment, multiple-week recovery). Figures 1–4 present data from the PD70 intervention group, allowed to age to PD98. Four genotypes of mice were studied: *Grin1*^+/+^ (WT), *Grin1*^+/+^:CreTg (WTCre), *Grin1*^flneo/flneo^ (*Grin1*^*KD*^), and *Grin1*^flneo/flneo^:CreTg (*Grin1*^*RESCUE*^). We determined that WT and WTCre mice had similar behavioural phenotypes in all of the subsequent studies (Supplementary Fig. 2), and thus experimental results for WT, *Grin1*^*KD*^ and *Grin1*^*RESCUE*^ mice were compared. Studies were performed with both male and female mice of equal number and powered to study the effect of sex.

We determined the extent of molecular recovery of *Grin1* mRNA and GluN1 protein by fluorescence in situ hybridisation and immunofluoresence (Fig. 1c), and the regional levels of NMDAR by [^3^H]MK-801 radioligand binding (Supplementary Fig. 3). Notable for our experimental objective, we observed substantial rescue of *Grin1* mRNA and NMDAR protein complex in the prefrontal cortex (PFC) of *Grin1*^*RESCUE*^ mice (Fig. 1c and Supplementary Fig. 3), affording the ability to test whether cognitive functions could recover from developmental NMDAR deficiency.

We also asked whether recovery of *Grin1* mRNA expression was achieved in both glutamatergic and GABAergic neurons of the cortex (Fig. 2a, b). In WT mice, the levels of *Grin1* are similar between *Gad1*+ GABAergic and *Vglut1*+ glutamatergic cells. This is consistent with single-cell transcriptomics data reference atlases [16], which indicate that *Grin1* is normally expressed in both cell types in the adult cortex, with higher levels observed in *Vglut1*+ cells (Fig. 2c). *Grin1*^*KD*^ mice have decreased *Grin1* mRNA in both *Gad1*+ cells and *Vglut1*+ cells (Fig. 2a, b). In the *Grin1*^*RESCUE*^ mice, *Grin1* mRNA was generally increased in *Vglut1*+ cortical neurons (Fig. 2a) but mRNA increases were less consistent in *Gad1*+ neurons of adjacent sections (Fig. 2b).

Since Cre recombination efficiency is affected by chromatin structure [17], we next queried a cell-type specific ATAC-seq database to determine whether the *Grin1* locus was more accessible in glutamatergic than GABAergic neurons of the mouse cortex [18]. Chromatin structure of the *Grin1* locus is indeed more accessible in glutamatergic neurons than in GABAergic neurons (Fig. 2d). Thus, we propose that the more consistent recovery of *Grin1* mRNA in glutamatergic neurons of the cortex reflects the more available chromatin structure at *Grin1* in these cells.

### NMDAR currents and synaptic GluN1 protein are restored in mPFC neurons

The extent of functional NMDAR recovery in the cortex was determined through whole cell electrophysiological recordings from brain slices. Physiological recordings from layer V pyramidal neurons of medial PFC (mPFC) were performed (Fig. 3a). Bath applied NMDA elicited an inward current in wild-type cells. This current was greatly attenuated in *Grin1*^*KD*^ mice; however, in *Grin1*^*RESCUE*^ mice, NMDA-elicited current was restored to wild-type levels (Fig. 3b, c). The differences in functional NMDARs occurred in the presence of largely similar intrinsic membrane properties (Supplementary Table I). However, capacitance was significantly larger in prefrontal neurons of *Grin1*^*RESCUE*^ compared to WT mice (Fig. 3d). Accordingly, we analyzed the current density of the NMDA-elicited currents (Fig. 3e; effect of genotype, *F*_2,95_ = 3.6, *p* = 0.03) and found that *Grin1*^*RESCUE*^ mice also had greatly increased current density compared to *Grin1*^*KD*^ mice.

Non-NMDA glutamate receptors in layer V neurons were examined by measuring spontaneous excitatory post-synaptic potentials (sEPSCs) under conditions that preclude NMDAR opening (holding potential of −75 mV with 2 mM Mg^2+^). There was no difference in the amplitude of sEPSCs in slices from the three genotypes of mice, indicating similar levels of functioning for AMPA and kainate receptors (Supplementary Fig. 4). There was, however, an increase in the frequency of sEPSCs in *Grin1*^*KD*^ neurons. The elevated synaptic input of *Grin1*^*KD*^ layer V neurons was normalised in *Grin1*^*RESCUE*^ mice, suggesting an improvement in E/I balance (Supplementary Fig. 4).

As further demonstration of synaptic NMDAR recovery in the PFC, the levels of GluN1 protein were determined by immunoprecipitation with anti-PSD-95 antibody and mass spectrometry. This procedure isolates the proteins that are part of the PSD-95 post-synaptic protein complex. Specifically, the amount of GluN1 peptide IVNIGAVLSTR was determined relative to the intensity of PSD-95 peptides, to indicate the abundance of GluN1 protein at PFC synapses. As shown in Fig. 3f, GluN1 peptide throughout the PFC was reduced in *Grin1*^*KD*^ mice and restored to wild-type levels in *Grin1*^*RESCUE*^ mice (WT: 0.79 ± 0.06, *Grin1*^*RESCUE*^: 0.67 ± 0.02, *p* = 0.077, power = 1.00).

### Cognitive impairments are rescued by adult intervention

We studied several domains of cognition that are used as endophenotypes for autism related neurodevelopmental disorders: habituation to a novel environment, sensory processing of acoustic startle, executive function, social interaction, and anxiety. Although each of these behaviours relies on more than cortical function for their performance, previous studies have repeatedly demonstrated the critical role that the PFC plays in these cognitive tasks. Indeed, cell-selective knockout of NMDARs in cortical neurons is sufficient to impair habituation, sensory processing of acoustic startle, social interaction, and anxiety [19–21].

Habituation to a novel environment requires the cortical and hippocampal processes of working and spatial memory to reduce exploration activity after a period of time [22, 23]. Habituation was quantified by calculating the habituation index (H.I.), which is the time required to reach half of the maximal locomotor activity using linear regression. *Grin1*^*KD*^ mice showed initial hyperactivity relative to WT in the first 10 min of exploration, and 120 min later, these mutant mice continued to explore the arena with high levels of activity (extrapolated H.I.: 149.1 ± 15.6 min; Fig. 4a–c). In contrast, while *Grin1*^*RESCUE*^ rescue mice also showed initial hyperactivity, their habituation to the novel environment was similar to WT mice (H.I.: WT 53.5 ± 1.2 min, *Grin1*^*RESCUE*^ 64.4 ± 3.2 min; Fig. 4a–c, *p* > 0.99, power = 1.00).

While assessing novelty-induced locomotion, we simultaneously measured stereotypy, an endophenotype of the repetitive behaviours that are observed in GRIN disorder. *Grin1*^*KD*^ mice display increased stereotypy that is 250% of WT levels (Fig. 4d). *Grin1*^*RESCUE*^ mice displayed only a modest improvement in stereotypy that is still 180% of WT (Fig. 4d), in contrast to the substantial improvements observed for habituation.

Sensorimotor gating, which is modulated by cortical arousal circuits [24], was measured with the paradigm of pre-pulse inhibition (PPI) of acoustic startle response (ASR). Consistent with studies of the original knockdown mutation [25], *Grin1*^*KD*^ mice exhibited deficits in sensorimotor gating at pre-pulse intensities of 4, 8, and 16 dB (Fig. 4e). This indicated that the pre-cognitive ability to attenuate motor response to a startling sound was impaired. *Grin1*^*RESCUE*^ mice showed a complete restoration of sensory processing in this test, with PPI levels that were similar to WT littermates (Fig. 4e, *p* > 0.99, power > 0.99). Interestingly, although PPI was normalised in the *Grin1*^*RESCUE*^ mice, the genetic intervention had little effect on the amplitude of the startle reflex itself. Both *Grin1*^*KD*^ and *Grin1*^*RESCUE*^ mice had a similar exaggeration in their startle amplitude that was 330% and 250% of WT, respectively (Fig. 4f).

Executive function was tested in the puzzle box test, which measured the ability of the mouse to overcome increasingly challenging obstacles and reach a goal box. Mice were first introduced to the arena with an open doorway leading to the goal, but on subsequent tests the doorway was blocked, and mice had to use an underpass or dig through bedding to reach the goal. Thus, the test measured goal-directed behaviour and cognitive flexibility to respond to different challenges [26]. *Grin1*^*KD*^ mice performed markedly worse than WT in early trials, taking 4–5 times longer to reach the goal box, and routinely failed the most challenging task of digging through bedding (Fig. 4g). Impressively, *Grin1*^*RESCUE*^ mice solved both challenges, and performed significantly better than *Grin1*^*KD*^ mice on all trials (Fig. 4g). Indeed, in three of the seven trials, *Grin1*^*RESCUE*^ mice performed similar to WT mice. Thus, there were substantial improvements in executive function as assessed in the puzzle box test.

Affiliative social behaviour was studied by measuring the amount of time that a mouse spent investigating a novel C57Bl/6J mouse. The novel mouse was constrained in one area with a wire cage, and an empty cage was included in the arena to control for non-social investigation of the cage. As expected, social interaction was significantly impaired in *Grin1*^*KD*^ mice relative to WT, controlling for locomotor activity (Fig. 4h). *Grin1*^*RESCUE*^ mice displayed social interaction that was completely restored to WT levels (*p* > 0.99, power = 1.00). Not only was the amount of time spent in social interaction normalised in *Grin1*^*RESCUE*^ mice, but the quality of social interaction appeared to improve, as demonstrated by the longer time spent with each visit to the novel mouse (Fig. 4i). While there were genotype differences in the amount of time spent in social interaction, all three genotypes of mice showed a similar preference for social investigation over non-social investigation of the empty cage, as reflected in a similar discrimination index between genotypes (Fig. 4j). Thus the improvement in social interaction reflected an improved quality of social interaction rather than a change in social motivation.

Lastly, we measured anxiety-like behaviour in the elevated plus maze. WT mice spent less than 20% of time in the open arms of the maze; in contrast, *Grin1*^*KD*^ mice spent nearly 100% of time in the open arms (Fig. 4k). In this behavioural domain, the *Grin1*^*RESCUE*^ mice showed an intermediate phenotype, spending 50% of time in the open arms of the maze. The amount of time spent in the open arms by each genotype is further confirmed in the number of entries observed into the open arm (Fig. 4l). The three genotypes had a similar number of total arm entries (Fig. 4l), and ANCOVA analysis to control for differences in locomotor activity still showed a significant effect of genotype.

We observed similar patterns of behavioural deficits in male and female *Grin1*^*KD*^ mice, and similar patterns of recovery in *Grin1*^*RESCUE*^ male and female mice in most tests. There were notable sex differences in a select behavioural test, the puzzle box test: for all three genotypes, female mice performed better than male mice of the same genotype (Supplementary Fig. 5).

It should also be noted that, in the puzzle box test, the female *Grin1*^*RESCUE*^ mice showed a greater improvement than male *Grin1*^*RESCUE*^ mice (Supplementary Fig. 5).

In summary, our battery of behavioural tests pointed to the most effective rescue of cognitive functions that included habituation to novelty, sensorimotor gating, executive function, and social investigation. Intermediate levels of rescue were observed for the initial hyperlocomotor response to novelty and anxiety-like behaviour. Minimal rescue was observed for stereotypy and the acoustic startle reflex response.

### Cognitive improvements persist with a longer recovery period

**Fig. 5:**
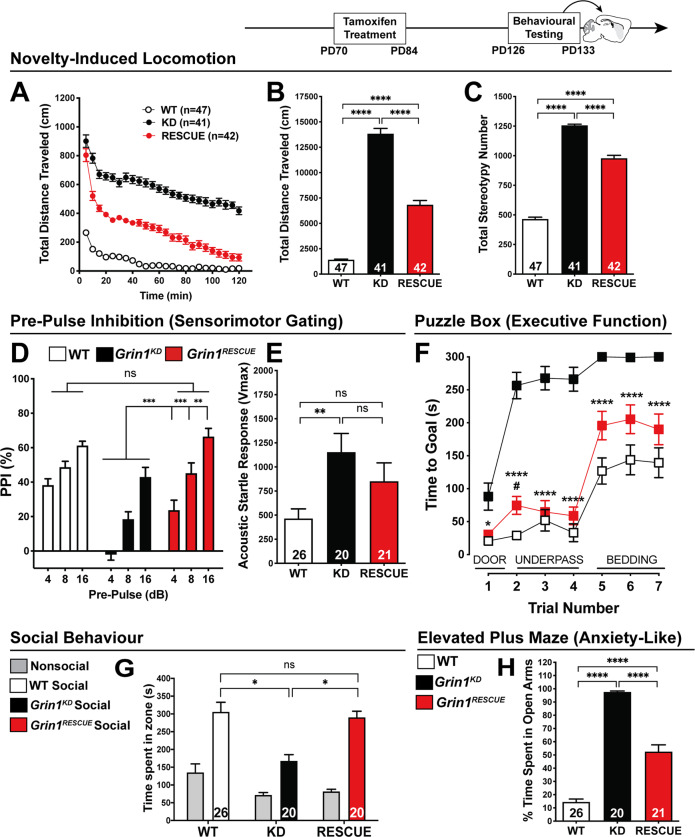
Replicable and robust cognitive function improvement with adult intervention (PD70) and extended recovery time. All behaviours were measured in WT, *Grin1*^*KD*^, and *Grin1*^*RESCUE*^ mice starting at PD126. **a** Time-course and **b** total distance travelled (cm) in open field (OF) test. One-way ANOVA (total, effect of genotype *F*_2,127_ = 276.0, *p* < 0.0001), Bonferroni post hoc. **c** Stereotypy number in OF test. One-way ANOVA, effect of genotype, *F*_2,127_ = 506.7, *p* < 0.0001, Bonferroni post hoc. **d** Percent inhibition of startle response (pre-pulse inhibition, PPI). Shown on graph, *Grin1*^*KD*^ vs*. Grin1*^*RESCUE*^, two-way ANOVA, effect of genotype—*F*_2,64_ = 15.81, *p* < 0.0001; genotype × decibel—*F*_4,128_ = 7.060, *p* < 0.0001, Bonferroni post hoc. **e** Acoustic startle response (ASR). One-way ANOVA, effect of genotype, *F*_2,64_ = 4.873, *p* = 0.0107, Bonferroni post hoc. **f** Time to reach goal zone (seconds; max 300 s) measured in puzzle box paradigm. Shown on graph, ^#^WT vs. *Grin1*^*RESCUE*^; *Grin1^*KD*^ vs. *Grin1*^*RESCUE*.^ Two-way ANOVA, effect of genotype: *F*_2,61_ = 60.36, *p* < 0.0001, Bonferroni post hoc. *n* values: WT—21, *Grin1*^*KD*^—21, *Grin1*^*RESCUE*^—22. **g** Total time spent in zones during 10 min. modified three chamber social test. One-way ANCOVA, there is an effect of genotype after controlling for locomotor activity on time spent in the social zone, *F*_2,63_ = 4.061, *p* = 0.022, Bonferroni post hoc. **h** % time spent in open arms in the elevated plus maze (EPM). One-way ANCOVA, there is an effect of genotype on % time spent in open arms after controlling for locomotor activity, *F*_2,63_ = 156.537, *p* < 0.001, Bonferroni post hoc. All data shown as mean ± SEM, *p < 0.05; **p < 0.01; ***p < 0.001; ****p < 0.0001, ns not significant.

Finally, we asked whether these behavioural improvements would persist or would further improve with a longer recovery period. We also hypothesised that a longer recovery period might be necessary for those behaviours that were not robustly improved after only 2 weeks. Therefore, in a distinct cohort of experimental and control mice, we induced Cre-mediated rescue of *Grin1* at PD70, as in the original paradigm, but waited an additional 4 weeks before testing the animals (6-week recovery vs. original 2-week recovery). In this cohort, there was a similar degree of rescue in the level of NMDARs (Supplementary Table II). Behaviourally, the *Grin1*^*RESCUE*^ mice showed significant improvement across all measures examined (Fig. 5), in a pattern consistent with the assessments presented in Fig. 4. *Grin1*^*RESCUE*^ mice, treated at PD70, and allowed to recover for 6 weeks, showed improvement in their sensorimotor gating (PPI, Fig. 5d), executive function (EF, Fig. 5f), and affiliative social behaviour (AS, Fig. 5g), that was similar to WT (PPI: (4 dB) *p* = 0.072, power = 1.00, (8 dB) *p* = 1.00, power = 0.99, (16 dB) *p* = 1.00, power = 0.94; EF: *p* > 0.05, power = 1.00; AS: *p* > 0.99, power = 0.99). These experiments indicate that adult intervention leads to sustained cognitive improvement that can be observed as early as 2 weeks after completion of treatment.

**Fig. 6:**
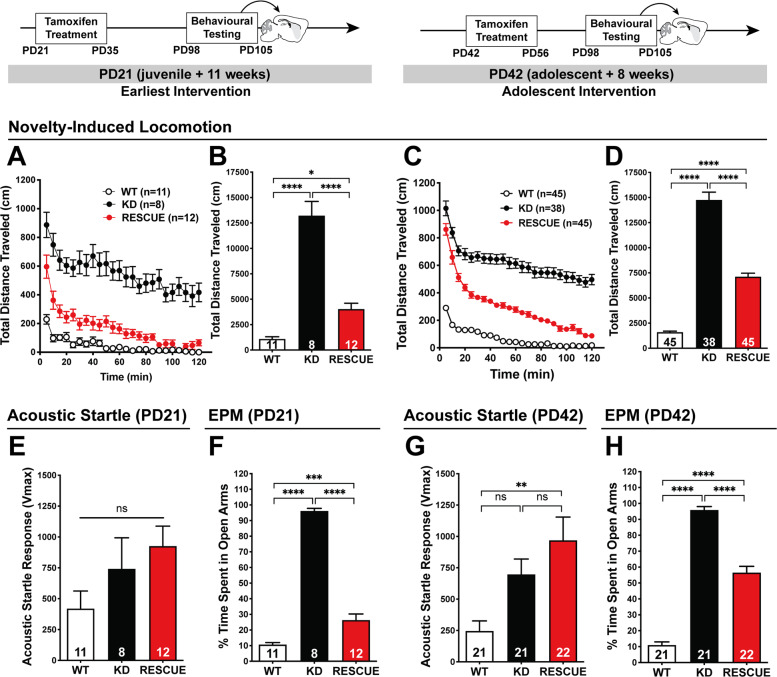
Effect of early intervention on behaviours that are rescue-refractory with adult intervention. All behaviours were measured in WT, *Grin1*^*KD*^, and *Grin1*^*RESCUE*^ mice starting at PD126, following intervention at either PD21 (left), or PD42 (right). **a**, **c** Time-course and **b**, **d** total distance travelled (cm) in open field (OF) test. One-way ANOVA, total distance travelled, effect of genotype: (PD21) *F*_2,28_ = 62.53, *p* < 0.0001, (PD42) *F*_2,125_ = 202.7, *p* < 0.0001, Bonferroni post hoc. **e**, **g** Acoustic startle response (ASR). One-way ANOVA, effect of genotype, (PD21) *F*_2,28_ = 2.249, *p* = 0.1242, (PD42) *F*_2,61_ = 6.975, *p* = 0.0019, Bonferroni post hoc. **f**, **h** Percent time spent in open arms in the elevated plus maze (EPM). One-way ANOVA, effect of genotype, (PD21) *F*_2,28_ = 219.1, *p* < 0.0001, (PD42) *F*_2,61_ = 210.9, *p* < 0.0001, Bonferroni post hoc. All data shown as mean ± SEM, *p < 0.05; **p < 0.01; ***p < 0.001; ****p < 0.0001, ns not significant.

There were also indications of sustained improvements in overall health, since deficits in body mass were normalised after 6 weeks of recovery (Supplementary Fig. 6). However, the longer recovery period did not provide greater levels of improvement in “rescue-refractory” behaviours: initial locomotor hyperactivity (Fig. 5a, b), stereotypy (Fig. 5c), ASR (Fig. 5e), or anxiety-like behaviour (Fig. 5h). Therefore, we also conducted experiments where genetic rescue was initiated at earlier stages of development, focusing on some of the “rescue-refractory” behaviours to determine whether earlier stages of intervention were necessary. The same tamoxifen administration regimen was given to mice at 3- and 6- weeks of age (PD21 or PD42), and the mice were allowed to age until PD98 (Fig. 6). There was no benefit to earlier treatment at PD42 in domains of locomotor hyperactivity, acoustic startle, or anxiety (Fig. 6c, d, g, h; interaction of genotype × intervention: (locomotor) *F*_2,251_ = 1.704, *p* = 0.184, (acoustic startle) *F*_2,121_ = 3.001, *p* = 0.053, (EPM) *F*_2,121_ = 2.028, *p* = 0.136, post hoc showing no difference between interventions within *Grin1*^*RESCUE*^, *p* > 0.05). However, treatment at PD21 did provide more substantial improvements in anxiety-like behaviours (Fig. 6f), when compared to adult intervention (PD70; Fig. 4k) (interaction of genotype × intervention: *F*_1,86_ = 18.631, *p* < 0.001), and post hoc analysis showed this was significant for the *Grin1*^*RESCUE*^ (*p* < 0.001) but not the *Grin1*^*KD*^ or WT (*p* = 1.000 for both).

## Discussion

The knockdown of *Grin1* results in viable mutant mice with deficits in cognitive behaviours that parallel the symptoms of *GRIN1* encephalopathy [13]. We did not find evidence of any deleterious effects from the postnatal upregulation of NMDARs. *Grin1*^*RESCUE*^ mice had healthier coats, reached normal body weights, and were less reactive to handling after Cre induction. We found improvements in nearly every aspect of behaviour that we examined.

Our strategy to achieve temporal rescue of NMDARs took advantage of a tamoxifen-inducible Cre recombinase [27]. The study design allowed us to treat all groups of mice with tamoxifen, reducing the likelihood that the behavioural recovery of *Grin1*^RESCUE^ mice would be obscured by the drug treatment. Vogt et al. showed that a 4-week washout period was sufficient to avoid tamoxifen’s effects on cognition [28]. We observed that the biochemical and behavioural measures were remarkably similar with a 2- or 6-week washout (Figs. 4, 5, Supplementary Fig. 3, Table II), suggesting that tamoxifen had little effect on our measures of recovery.

This study focused primarily on the question of whether or not any recovery of neurodevelopmental deficits was possible, and if so, when in development must an intervention occur. Our results show that remarkable recovery is possible in adulthood and that similar outcomes occur in both the adolescent and adult brain. Future studies will determine whether recovery leads to a replenishment of white matter volumes and synapse number, since NMDAR deficient mice have white matter deficits [29] and reduced synapse density in the cortex and striatum [30, 31]. Studies of the molecular and cellular events that occur with recovery could provide insight into the means by which the brain rewires and recovers from a neurodevelopmental insult.

One limitation to the full recovery of all behavioural abnormalities was the cellular and regional differences in the normalisation of *Grin1* mRNA that were achieved in *Grin1*^*RESCUE*^ mice. Indeed, the behaviours associated with striatum function, such as hyperactivity and stereotypy, were not completely normalised in *Grin1*^*RESCUE*^ mice. This is likely due to the limited increase in *Grin1* mRNA and NMDAR function in that brain region (Supplementary Fig. 7, Table II, III). Even within the cortex, we observed variability in the levels of rescue. We noted in *Grin1*^*RESCUE*^ mice that glutamatergic cells, which have a more open chromatin structure at *Grin1*, had a more consistent expression of *Grin1* than GABAergic cells, which have a more closed chromatin structure at that locus. Therefore, we hypothesise that regional and cellular differences in recovery are influenced by chromatin accessibility, which should be considered in the context of future gene-editing therapies.

In spite of these limitations, our results provide striking evidence of the plasticity of the adult brain, particularly in the cortex. Within the cortex the highest levels of recovery were observed in *Vglut1*+ cells, which normally express the highest levels of *Grin1* [16]. It is possible that very early interventions would provide a more complete recovery in some cell types or brain functions. However, our results suggest that symptoms of intellectual disability, a consistent symptom of *GRIN* disorder [4], can be largely treated with adult intervention. This is particularly surprising since cognitive impairments are refractory to current pharmacological treatment in patients with autism and schizophrenia [32], two conditions associated with impaired NMDAR function [33]. Adult genetic reversal has an even greater clinical impact, as it offers the possibility of stable restoration of normal function even after the brain has completed development [34, 35].

The prevalence of pathogenic variants has been estimated at 5.45 per 100,000 births for *GRIN1*, and 3.23 and 5.91 per 100,000 for *GRIN2A* and *GRIN2B* respectively [36, 37]. The first patients to be sequenced had diagnoses of intellectual disability [38] or epilepsy [13]. A recent whole-exome sequencing study reported that 7% of patients with autism or schizophrenia carry a predicted-deleterious coding mutation in one of six *GRIN* genes (25/370 patients with schizophrenia, 15/192 patients with autism) [39]. There have also been numerous genetic and epidemiological studies supporting a causal role for NMDARs in several neuropsychiatric disorders. Thus, the significance of our findings is not limited to those patients who have been sequenced to date.

This study highlights the significant potential of therapeutic intervention in adult patients. It demonstrates that a delay between symptom onset and treatment can be overcome. The cognitive symptoms of neuropsychiatric and neurodevelopmental conditions caused by NMDAR hypofunction are amenable to treatment and show persisting improvement. The mature cortex has sufficient plasticity to recover from insults to this key developmental system, and adult intervention with the appropriate therapeutic agent should treat intellectual disabilities.

## Materials and methods

### Animals

Animal housing and experimentation were carried out in accordance with the Canadian Council in Animal Care guidelines for the care and use of animals and following protocols approved by the Faculty of Medicine and Pharmacy Animal Care Committee at the University of Toronto. Mice were group housed with littermates on 12 h light–dark cycle and were given ad libitum access to water and food.

*ROSA26*^*CreERT2*^ mice were obtained from Jackson Laboratory (008463; B6.129-*Gt(ROSA)26Sor*^*tm1(cre/ERT2)Tyj*^*/J*), and were previously described [27]. The Cre-reporter mouse line used, *ROSA26*^*tdTomato*^, was obtained from Jackson Laboratory (007914; B6.Cg-*Gt(ROSA)26Sor*^*t*m*14(CAG-tdTomato)Hze*^/J) [40] and was crossed with the *ROSA26*^*CreERT2*^ line.

*Grin1*^*flneo/flneo*^ mice were generated at the University of Toronto, based on the previously described *Grin1*^*neo/neo*^ mouse [15]. Identical to the *Grin1*^*neo/neo*^ model, the *Grin1* gene was modified via homologous recombination with an intervening sequence (neomycin cassette), and targeted into intron 19, flanked by *loxP* sites (pXena vector; gift of Dr. Beverly Koller).

*Grin1*^*+/flneo*^:CreTg mice were produced by crossing *ROSA26*^*CreERT2*^ C57Bl/6J congenic mice to *Grin1*^*+/flneo*^ C57Bl/6J congenics. The resulting compound heterozygotes were bred to *Grin1*^*+/flneo*^ 129/SvlmJ congenics to produce the F1 progeny used for all experiments as recommended by the Banbury Conference [41]. Experimental mice of the F1 background were: *Grin1*^*+/+*^ (WT), *Grin1*^*+/+*^*:*CreTg (WTCre), *Grin1*^*flneo/flneo*^ (*Grin1*^*KD*^), and *Grin1*^*+/flneo*^:CreTg (*Grin1*^*RESCUE*^).

### Tamoxifen administration

Tamoxifen was administered to all genotypes of mice (WT, WTCre, *Grin1*^*KD*^, *Grin1*^*RESCUE*^). Tamoxifen (T5648, Sigma-Aldrich, St. Louis, MO, USA) was administered via oral gavage (6 mg, 20 mg/ml dissolved in 100% corn oil at 65 °C for 1 h) on day 1 of treatment, and then mice were given tamoxifen chow (TD.140425, 500 mg/kg, Envigo) ad libitum for 14 days.

### Behavioural testing

Male and female mice of equal numbers were used for behavioural testing. Tests were administered at PD98 or PD126. All experimental animals were first tested for locomotor activity on day 1. Mice were then assigned to one of two groups for subsequent behavioural tests that spanned 3 days. The puzzle box test [26] was administered to mice in Group A over days 2–4. Mice in Group B were tested in elevated plus maze on day 2, social affiliative paradigm on day 3, and PPI of acoustic startle on day 4, as previously described [42–45].

### Fluorescent in situ hybridisation

Expression of *Grin1, Vglut1, and Gad1* mRNA in WT, *Grin1*^*KD*^, and *Grin1*^*RESCUE*^ mice was visualised by RNAscope Multiplex Fluorescent Reagent Kit v2 protocol (ACD Bio; CA, USA). Fresh frozen mouse brains were used to collect 20 µm sagittal sections (1.2 and 1.53 mm from midline). Sections were hybridised to *Grin1* probes (#533691-C1, ACD Bio) and *Vglut1* probes (#416631-C2), to *Grin1* probes and *Gad1* probes (#400951-C3) or to control probe mixtures (positive control probes #320881, negative control probes #320871). Processed slides were imaged at 20X magnification with an Axio Scan.Z1 slide scanner (Zeiss, Oberkochen, DEU) or 40X magnification with an AxioObserverZ1 Inverted Motorised Microscope.

### Re-analysis of publicly-accessible single-cell and cell type-specific genomics data

We obtained single-cell RNAseq data sampled from the adult mouse visual cortex via the Allen Institute for Brain Science’s Cell Types database (http://celltypes.brain-map.org/) [16] and pooled cell type-specific ATACseq (Assay for Transposase-Accessible Chromatin) data sampled by the Allen Institute for Brain Sciences from the adult mouse visual cortex from the Gene Expression Omnibus repository (GSE87548) [18].

### GluN1 immunofluorescent visualisation

Expression of GluN1 protein levels in WT, *Grin1*^*KD*^, and *Grin1*^*RESCUE*^ mice were on fresh frozen sagittal tissue sections (20 µm thick; Lateral ~1.92 mm). Sections were incubated with an in-house rabbit anti-GluN1 antibody raised against peptide ETEKPRGYQMSTRLK (C) (1:200), and then with secondary antibody, anti-rabbit Alexa 568 (ThermoFisher, #A11011, 1:500).

### [^3^H]MK-801 saturation binding

NMDAR levels in WT, *Grin1*^*KD*^, and *Grin1*^*RESCUE*^ mice were quantified in prefrontal cortical and striatal tissue. The following solutions were prepared: membranes, 1.6 µg/µl working concentration; [^3^H]MK-801 (Perkin Elmer), 120 nM working concentration; and cold MK-801 (Sigma-Aldrich), 1200 nM working solution (10× [^3^H]MK-801). Binding assays were performed with the NMDAR antagonist MK-801 (hot and/or cold), mouse brain membranes (80 µg) and binding buffer (total binding vs. non-specific binding), with a total assay volume of 150 µl. Radioactivity was quantified via liquid scintillation spectrometry [46].

### PSD-95 immunoprecipitation mass spectrometry

As previously described [47], mouse anti-PSD-95 antibody (Millipore, catalogue # MAB1596) was used to capture PSD-95 protein complexes from flash frozen cortex samples (3 males and 3 females of each genotype were used). 5 μg of PSD-95 antibody was coupled per 1 mg of Dynabeads (Life Technologies; antibody coupling kit protocol (#14311D). The data was recorded using Analyst-TF (version 1.7) software and analyzed by Sciex DIA software to generate peptide intensities.

### Electrophysiological recordings

Coronal slices (400 µm) of the mPFC (1.98–1.34 mm [12]) and caudate putamen (1.54–0.14 mm) were used. Most experiments were performed in the presence of CNQX disodium salt (20 µm; Alomone Labs) to block AMPA receptors. NMDA (30 µm; Sigma-Aldrich) was bath applied. Application of APV (50 µM; Alomone Labs) confirmed the inward currents were mediated by NMDARs. Peak amplitude of the NMDA currents was measured using Clampfit software (Molecular Devices). Magnitude of NMDA-elicited inward currents was quantified by subtracting a 1 s average holding current at the peak from the average holding current at the baseline.

### Quantification and statistical analysis

Statistically significant outliers were calculated and excluded, using the Grubb’s Test. Data were analyzed either using a one- or two-way ANOVA (repeated measures), or one-way ANCOVA where indicated, with multiple comparisons and post-hoc Bonferroni’s test, as indicated in figure legends. For electrophysiological recordings, paired *t* tests were used to compare neuronal responses to NMDA before and after APV. Data analysis was not blinded. For single-cell RNAseq and cell-type specific ATACseq results, data was compared using Wilcoxon rank-sum tests. Differences in means were considered statistically significant at *p* < 0.05. Significance levels are as follows; *p < 0.05; **p < 0.01; ***p < 0.001; ****p < 0.0001, ns—not significant. All data analyses were performed using the Graphpad Prism 6.0 software and/or IBM SPSS 23.0 Software and using custom analysis scripts written in R.

## Supplementary information

Supplemental methods figures tables and legends

## References

[1] Maulik PK, Mascarenhas MN, Mathers CD, Dua T, Saxena S (2011). Prevalence of intellectual disability: a meta-analysis of population-based studies. Res Dev Disabil.

[2] Fombonne E (2009). Epidemiology of pervasive developmental disorders. Pediatr Res..

[3] Mohammed S, Scott R, Vogt J, Al-Turki S, Cross G, Smithson S (2014). Large-scale discovery of novel genetic causes of developmental disorders. Nature..

[4] XiangWei W, Jiang Y, Yuan H (2018). De novo mutations and rare variants occurring in NMDA receptors. Curr Opin Physiol.

[5] Guy J, Gan J, Selfridge J, Cobb S, Bird A (2007). Reversal of neurological defects in a mouse model of Rett syndrome. Science.

[6] Mei Y, Monteiro P, Zhou Y, Kim J-AA, Gao X, Fu Z (2016). Adult restoration of Shank3 expression rescues selective autistic-like phenotypes. Nature..

[7] Li Y, Erzurumlu RS, Chen C, Jhaveri S, Tonegawa S (1994). Whisker-related neuronal patterns fail to develop in the trigeminal brainstem nuclei of NMDAR1 knockout mice. Cell..

[8] Suzuki A, Lo F-S, Zhao S, Itohara S, Hayashi Y, Arakawa H (2014). Thalamic NMDA receptor function is necessary for patterning of the thalamocortical somatosensory map and for sensorimotor behaviors. J Neurosci..

[9] Iwasato T, Datwani A, Wolf AM, Nishiyama H, Taguchi Y, Tonegawa S (2000). Cortex-restricted disruption of NMDAR1 impairs neuronal patterns in the barrel cortex. Nature..

[10] Gu X, Zhou L, Lu W (2016). An NMDA receptor-dependent mechanism underlies inhibitory synapse development. Cell Rep..

[11] Zhang Z-w, Peterson M, Liu H (2013). Essential role of postsynaptic NMDA receptors in developmental refinement of excitatory synapses. Proc Natl Acad Sci.

[12] Espinosa JS, Wheeler DG, Tsien RW, Luo L (2009). Uncoupling dendrite growth and patterning: single-cell knockout analysis of NMDA receptor 2B. Neuron..

[13] Lemke JR, Geider K, Helbig KL, Heyne HO, Schütz H, Hentschel J (2016). Delineating the GRIN1 phenotypic spectrum: a distinct genetic NMDA receptor encephalopathy. Neurology..

[14] Forrest D, Yuzaki M, Soares HD, Ng L, Luk DC, Sheng M (1994). Targeted disruption of NMDA receptor 1 gene abolishes NMDA response and results in neonatal death. Neuron..

[15] Mohn AR, Gainetdinov RR, Caron MG, Koller BH (1999). Mice with reduced NMDA receptor expression display behaviors related to schizophrenia. Cell..

[16] Tasic B, Yao Z, Graybuck LT, Smith KA, Nguyen TN, Bertagnolli D (2018). Shared and distinct transcriptomic cell types across neocortical areas. Nature..

[17] Long MA, Rossi FMV. Silencing inhibits cre-mediated recombination of the Z/AP and Z/EG reporters in adult cells. PLoS ONE. 2009;4:1–8. 10.1371/journal.pone.0005435 PMC2672169 19415111

[18] Gray LT, Yao Z, Nguyen TN, Kim TK, Zeng H, Tasic B. Layer-specific chromatin accessibility landscapes reveal regulatory networks in adult mouse visual cortex. Elife. 2017;6:e21883. 10.7554/eLife.21883 PMC5325622 28112643

[19] Rompala GR, Zsiros V, Zhang S, Kolata SM, Nakazawa K (2013). Contribution of NMDA receptor hypofunction in prefrontal and cortical excitatory neurons to schizophrenia-like phenotypes. PLoS ONE..

[20] Belforte JE, Zsiros V, Sklar ER, Jiang Z, Yu G, Li Y (2010). Postnatal NMDA receptor ablation in corticolimbic interneurons confers schizophrenia-like phenotypes. Nat Neurosci..

[21] Finlay JM, Dunham GA, Isherwood AM, Newton CJ, Nguyen TV, Reppar PC (2015). Effects of prefrontal cortex and hippocampal NMDA NR1-subunit deletion on complex cognitive and social behaviors. Brain Res..

[22] Yamaguchi S, Hale LA, D’Esposito M, Knight RT (2004). Rapid prefrontal-hippocampal habituation to novel events. J Neurosci..

[23] Ranganath C, Rainer G (2003). Cognitive neuroscience: neural mechanisms for detecting and remembering novel events. Nat Rev Neurosci.

[24] Li L, Du Y, Li N, Wu X, Wu Y (2009). Top-down modulation of prepulse inhibition of the startle reflex in humans and rats. Neurosci Biobehav Rev.

[25] Duncan GE, Moy SS, Perez A, Eddy DM, Zinzow WM, Lieberman JA (2004). Deficits in sensorimotor gating and tests of social behavior in a genetic model of reduced NMDA receptor function. Behav Brain Res.

[26] Ben Abdallah NM-B, Fuss J, Trusel M, Galsworthy MJ, Bobsin K, Colacicco G (2011). The puzzle box as a simple and efficient behavioral test for exploring impairments of general cognition and executive functions in mouse models of schizophrenia. Exp Neurol..

[27] Ventura A, Kirsch DG, McLaughlin ME, Tuveson DA, Grimm J, Lintault L (2007). Restoration of p53 function leads to tumour regression in vivo. Nature..

[28] Vogt MAA, Chourbaji S, Brandwein C, Dormann C, Sprengel R, Gass P (2008). Suitability of tamoxifen-induced mutagenesis for behavioral phenotyping. Exp Neurol.

[29] Intson K, van Eede MC, Islam R, Milenkovic M, Yan Y, Salahpour A, et al. Progressive neuroanatomical changes caused by Grin1 loss-of-function mutation. Neurobiol Dis. 2019;132:104527. 10.1016/j.nbd.2019.104527 31299220

[30] Chen Y, Milenkovic M, Horsfall W, Salahpour A, Soderling SH, Ramsey AJ (2018). Restoring striatal WAVE-1 improves maze exploration performance of GluN1 knockdown mice. PLoS ONE..

[31] Ramsey AJ, Milenkovic M, Oliveira AF, Escobedo-Lozoya Y, Seshadri S, Salahpour A (2011). Impaired NMDA receptor transmission alters striatal synapses and DISC1 protein in an age-dependent manner. Proc Natl Acad Sci USA.

[32] Millan MJ, Agid Y, Brüne M, Bullmore ET, Carter CS, Clayton NS (2012). Cognitive dysfunction in psychiatric disorders: Characteristics, causes and the quest for improved therapy. Nat Rev Drug Discov.

[33] Tarabeux J, Kebir O, Gauthier J, Hamdan FF, Xiong L, Piton A (2011). Rare mutations in N-methyl-D-aspartate glutamate receptors in autism spectrum disorders and schizophrenia. Transl Psychiatry..

[34] Van Duyne G. D. Cre recombinase. Microbiol. Spectr. 3:MDNA3-0014-2014. 10.1128/microbiolspec.MDNA3-0014-2014. 2015;119–38. 10.1128/microbiolspec.MDNA3-0014-2014 26104563

[35] Speed HE, Kouser M, Xuan Z, Liu S, Duong A, Powell CM. Apparent genetic rescue of adult shank3 exon 21 insertion mutation mice tempered by appropriate control experiments [published correction appears in eNeuro. 2020 Mar 9;7(2):]. eNeuro. 2019;6(5):ENEURO.0317-19.2019. Published 2019 Sep 27. 10.1523/ENEURO.0317-19.2019. 10.1523/ENEURO.0317-19.2019 PMC6774147 31451607

[36] López-Rivera JA, Pérez-Palma E, Symonds J, Lindy AS, McKnight DA, Leu C, et al. A catalogue of new incidence estimates of monogenic neurodevelopmental disorders caused by de novo variants. Brain. 2020;143:1099–1105. 10.1093/brain/awaa051. 10.1093/brain/awaa051 PMC7174049 32168371

[37] Lemke J. Predicting incidences of neurodevelopmental disorders. Brain. 2020:1046–8. https://www.ncbi.nlm.nih.gov/pubmed/?term=32318731. Accessed 4 May 2020. 10.1093/brain/awaa079 32318731

[38] Hamdan FF, Gauthier J, Araki Y, Lin DT, Yoshizawa Y, Higashi K (2011). Excess of de novo deleterious mutations in genes associated with glutamatergic systems in nonsyndromic intellectual disability. Am J Hum Genet.

[39] Yu Y, Lin Y, Takasaki Y, Wang C, Kimura H, Xing J (2018). Rare loss of function mutations in N-methyl-d-aspartate glutamate receptors and their contributions to schizophrenia susceptibility. Transl Psychiatry..

[40] Madisen L, Zwingman TA, Sunkin SM, Oh SW, Zariwala HA, Gu H (2010). A robust and high-throughput Cre reporting and characterization system for the whole mouse brain. Nat Neurosci.

[41] Silva AJ, Simpson EM, Takahashi JS, Lipp HP, Nakanishi S, Wehner JM (1997). Mutant mice and neuroscience: recommendations concerning genetic background. Neuron..

[42] Islam R, Trépanier M-O, Milenkovic M, Horsfall W, Salahpour A, Bazinet RP (2017). Vulnerability to omega-3 deprivation in a mouse model of NMDA receptor. Npj Schizophr.

[43] Mielnik CA, Horsfall W, Ramsey AJ (2014). Diazepam improves aspects of social behaviour and neuron activation in NMDA receptor-deficient mice. Genes Brain Behav.

[44] Milenkovic M, Mielnik CA, Ramsey AJ (2014). NMDA receptor-deficient mice display sexual dimorphism in the onset and severity of behavioural abnormalities. Genes Brain Behav.

[45] Moy SS, Nadler JJ, Young NB, Perez A, Holloway LP, Barbaro RP (2007). Mouse behavioral tasks relevant to autism: Phenotypes of 10 inbred strains. Behav Brain Res.

[46] DeBlasi A, O’Reilly K, Motulsky HJ (1989). Calculating receptor number from binding experiments using same compound as radioligand and competitor. Trends Pharmacol Sci.

[47] Sullivan CR, Mielnik CA, O’Donovan SM, Funk AJ, Bentea E, DePasquale EA, et al. Connectivity analyses of bioenergetic changes in schizophrenia: identification of novel treatments. Mol Neurobiol. 2018;56:4492–517. 10.1007/s12035-018-1390-4 PMC7584383 30338483

[48] Paxinos G, Franklin KBJ. The mouse brain in stereotaxic coordinates. 2nd edition, Academic Press, San Diego. 2001.

